# Combining dosiomics and machine learning methods for predicting severe cardiac diseases in childhood cancer survivors: the French Childhood Cancer Survivor Study

**DOI:** 10.3389/fonc.2024.1241221

**Published:** 2024-12-02

**Authors:** Mahmoud Bentriou, Véronique Letort, Stefania Chounta, Brice Fresneau, Duyen Do, Nadia Haddy, Ibrahima Diallo, Neige Journy, Monia Zidane, Thibaud Charrier, Naila Aba, Claire Ducos, Vincent S. Zossou, Florent de Vathaire, Rodrigue S. Allodji, Sarah Lemler

**Affiliations:** ^1^ Université Paris-Saclay, CentraleSupélec, Mathématiques et Informatique pour la Complexité et les Systèmes, Gif-sur-Yvette, France; ^2^ Université Paris-Saclay, Université Versailles - Saint Quentin en Yvelines (UVSQ), Institut national de la santé et de la recherche médicale (INSERM), CESP-U1018, Villejuif, France; ^3^ Institut national de la santé et de la recherche médicale (INSERM), CESP-U1018, Radiation Epidemiology Team, Villejuif, France; ^4^ Gustave Roussy, Department of Clinical Research, Radiation Epidemiology Team, Villejuif, France; ^5^ Gustave Roussy, Department of Pediatric Oncology, Villejuif, France; ^6^ Department of Radiation Oncology, Gustave Roussy, Paris, France; ^7^ Gustave Roussy, Institut national de la santé et de la recherche médicale (INSERM), Radiothérapie Moléculaire et Innovation Thérapeutique, Paris-Saclay University, Villejuif, France; ^8^ Institut national de la santé et de la recherche médicale (INSERM), U900, Institut Curie, PSL Research University, Saint-Cloud, France; ^9^ Polytechnic School of Abomey-Calavi (EPAC), University of Abomey-Calavi, Cotonou, Benin

**Keywords:** survival analysis, dosiomics, cardiac disease, childhood cancer, machine learning, FCCSS

## Abstract

**Background:**

Cardiac disease (CD) is a primary long-term diagnosed pathology among childhood cancer survivors. Dosiomics (radiomics extracted from the dose distribution) have received attention in the past few years to assess better the induced risk of radiotherapy (RT) than standard dosimetric features such as dose-volume indicators. Hence, using the spatial information contained in the dosiomics features with machine learning methods may improve the prediction of CD.

**Methods:**

We considered the 7670 5-year survivors of the French Childhood Cancer Survivors Study (FCCSS). Dose-volume and dosiomics features are extracted from the radiation dose distribution of 3943 patients treated with RT. Survival analysis is performed considering several groups of features and several models [Cox Proportional Hazard with Lasso penalty, Cox with Bootstrap Lasso selection, Random Survival Forests (RSF)]. We establish the performance of dosiomics compared to baseline models by estimating C-index and Integrated Brier Score (IBS) metrics with 5-fold stratified cross-validation and compare their time-dependent error curves.

**Results:**

An RSF model adjusted on the first-order dosiomics predictors extracted from the whole heart performed best regarding the C-index (0.792 ± 0.049), and an RSF model adjusted on the first-order dosiomics predictors extracted from the heart’s subparts performed best regarding the IBS (0.069 ± 0.05). However, the difference is not statistically significant with the standard models (C-index of Cox PH adjusted on dose-volume indicators: 0.791 ± 0.044; IBS of Cox PH adjusted on the mean dose to the heart: 0.074 ± 0.056).

**Conclusion:**

In this study, dosiomics models have slightly better performance metrics but they do not outperform the standard models significantly. Quantiles of the dose distribution may contain enough information to estimate the risk of late radio-induced high-grade CD in childhood cancer survivors.

## 1 Introduction

Improving childhood cancer care has resulted in an average 5-year survival rate up to 85% in high-income countries (1). Radiotherapy (RT) is an efficient cancer treatment that kills cancer cells and may be combined with other treatments such as chemotherapy. However, RT (2, 3) and chemotherapy (4) are known long-term risk factors for CDs (CD), one of childhood cancer survivors’ most diagnosed second pathologies and still underdiagnosed (3). Early prognosis of late effects of childhood cancer treatment is an important public health challenge that will allow better healthcare for survivors.

The standard method for the risk estimation of CD is based on statistical models (e.g. odd ratios, hazard ratios, excess relative risk) adjusted on the mean radiation dose received by the heart, or on metrics derived from the dose-volume histograms (5–10). Even if such indicators can be effective predictors, they do not consider the spatial heterogeneity of the dose distribution. Indeed, we know that delivered dose distributions in RT may have high dose variations within small distances (11). Therefore, statistical models might miss the effects related to such spatial heterogeneity.

When available, whole-body voxel-scale dosimetric data contains the spatial information of the dose distribution received by a patient during RT. At this point, there are two ways to use this information: either we use the 3D dose distribution as a raw input of any suitable predicting model (12) or preliminarily extract informative features from the dose distribution. In this study, we chose to explore the second one with dosiomics. Indeed, using well-defined features to represent the 3D dose distribution as predictors of our models makes them more explainable.

Dosiomics is a way to extract such informative features based on texture analysis techniques. This term has recently appeared in the literature and refers to radiomics applied over the 3D dose distribution of patients treated by RT (13, 14). Dosiomics takes into account more information about dose distribution, including spatial correlations. Their predictive power has been explored over several pathologies induced by RT, including radiation pneumonitis (15, 16), xerostomia (17), and rectal cancer (18), and is sometimes combined with radiomics extracted from CT images (14, 19, 20). Integrating the additional information of dosiomics compared to dose-volume histograms might improve the prognosis of CD. However, there is no clear evidence that such models would outperform standard statistical methods (14, 19). Note that other feature extraction methods based on deep learning representation are currently explored in the literature (21, 22).

Machine learning denotes specific advanced inference methods at the interface between computer science, statistics and optimization that have proven very efficient for classification or regression tasks. Going beyond their initial applications to classification or regression tasks, machine learning methods have been adapted to survival analysis (also called time-to-event analysis) (23, 24). However, selecting the best-performing machine learning method for a specific problem is still an open question (14, 25, 26).

This paper explores the application of machine learning methods using dosimetric features (mean dose, dose-volume indicators and dosiomics-based) for the prognosis of high-grade CD within the French Childhood Cancer Survivors Study (FCCSS), a large multi-centric cohort. The predictors are the dosimetric indicators extracted from the 3D voxelized dose distribution of the heart (including dosiomics), chemotherapy-related variables (a known factor of CD), and clinical variables. We perform survival analysis using standard Cox Proportional Hazard (27), Cox with Lasso penalty (24), Cox Bootstrap Lasso models (28), and Random Survival Forests (23) over several sets of features, including dosiomics or standard dosimetric predictors, to estimate the benefits of dosiomics and machine learning models. We also explore the benefits of extracting dosiomics over each heart’s subpart instead of the whole heart only. Efforts have been made to finely tune our machine-learning models and assess the statistical robustness of our results.

## 2 Materials and methods

### 2.1 Data

The French Childhood Cancer Survivors Study (FCCSS) is a large multi-centric cohort of 7670 patients diagnosed with cancer between 1946 and 2000, among five centers, before age 21, with a possible incomplete follow-up. In the FCCSS, 4197 patients have been treated by RT and whole-body voxelized dosimetric data were reconstructed for 3943 of them. The reconstruction of the 3D dose distribution is based on a voxel-based anthropomorphic phantom library (12 phantoms in total in this study) to generate a surrogate of the whole body as computed tomography (CT) image for each patient who received RT, with a voxel spacing of 2mm. Starting from twelve different patient anatomies (men and women of different ages), the algorithm produced an adjusted anatomy best matching the anatomy of each individual patient, taking into account the sex, age, and position adopted during radiotherapy, when this information was available (otherwise only gender and age were used) (29). Then, the RT beams, defined for each RT treatment of the patient, were mapped on the whole-body CT image. We refer to (30–32) for further descriptions of this method, previously applied in other studies.

We withdrew 300 patients with no available dose matrices (254 patients) or missing clinical and chemotherapy information (46 patients). Three additional patients were removed from the study because a CD occurred before their RT. Thus, our study integrates 7367 patients of the FCCSS, for whom 374 patients have experienced a CD with a grade above 3. We only consider high-grade CDs because CDs with lower grades are often self-declared, so that they could potentially induce a reporting bias. Since this work is based on a cohort study, with first diagnosed cancer year that spreads from 1946 to 2000, and high-grade CD is a late RT-induced risk, almost all of the patients have a right-censored survival time. Our analyses will have to take into account a large censorship rate (95%). The input of our analyses are: (i) the voxelized dose distribution received by the heart, which is segmented into subparts (left atrium, right atrium, left ventricle, right ventricle, myocardium), (ii) three clinical variables consisting of sex, age at diagnosis (categorized as 0-5 years, 6-10 years, 11-15 years, *>* 15 years), and type of the first diagnosed cancer; and (iii) two binary variables for chemotherapy: treatment involving anthracyclines or alkylating agents. The variable of interest to be predicted is the status (a high-grade CD has been diagnosed or not).

### 2.2 Feature extraction of 3D dose distribution

The 3D dose distribution data set is composed of 5181 files, where each file represents the dose distribution of a RT session. The mean and maximum number of voxels along each dimension of the heart’s 3D dose distributions are respectively (32, 42, 44) and (67, 70, 71). The voxel resolution is 2mm. A patient may correspond to several files because several RT sessions might be prescribed. In this case, dose distribution matrices are summed if the related treatments were executed within six months, beginning with the first RT treatment (above this threshold, the remaining treatments are untapped). We remove the outliers by thresholding the values greater than *D*_2_ (98% quantile of the dose distribution).

Dose-volume indicators and dosiomics were extracted from the voxelized dose distribution for each of five heart’s subpart (left atrium, right atrium, left ventricle, right ventricle, myocardium) or the whole heart. Dosiomics includes first-order statistics and texture indicators (including Gray Level Co-occurrence Matrix (GLCM), Gray Level Size Zone Matrix (GLSZM), Gray Level Run Length Matrix (GLRLM), Neighbouring Gray Tone Difference Matrix (NGTDM), and Gray Level Dependence Matrix (GLDM)). The chosen bin width for discretizing the histogram of doses is 0.5 Gy.

### 2.3 Statistical learning

**Figure 1:**
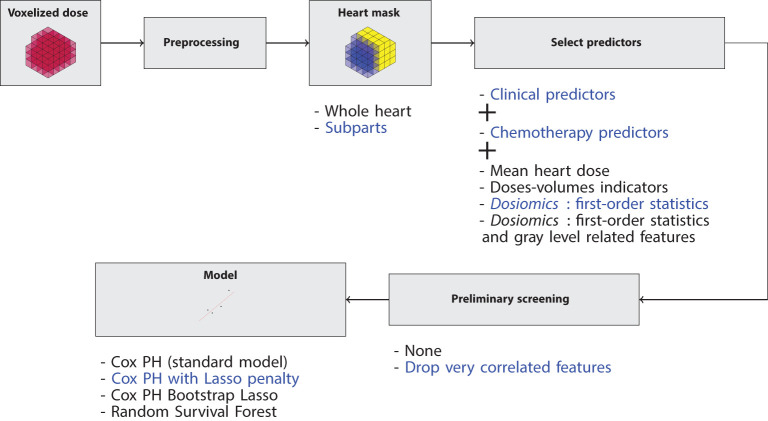
Workflow of the study. For each model, several options are possible: the heart can be considered a whole or a set of subparts; several groups of predictors can be considered; they can be preliminary filtered or not; four models can be used. We systematically explored each possible combination. For example, the blue path means that we compute the first-order dosiomics over each heart’s subpart, we perform the procedure described in Section 2.3.1, and we learn from the resulted predictors with a Cox Lasso model.

**Figure 1** summarizes the overall workflow of our study. After some preprocessing, we extract several groups of features and learn from these features the survival probabilities of the patients. Then, metrics are derived to select the best model and group of features, based on 5-fold stratified cross-validation as detailed further.

The high dimensionality and heterogeneity of the data raise several difficulties. In particular, it implies that several choices must be performed at each step: spatial scale (heart as a whole or considering its subparts), feature selection, preprocessing protocol and model types. In order to ensure that our conclusions were not biased by some particular choice, we explored systematically a large number of possible combinations for all these steps, as detailed hereafter.

#### 2.3.1 Preliminarily feature screening

The features are preliminary screened on the train set during each model fit of the cross-validation.

##### 2.3.1.1 Feature inclusion

For each model, the predictors include the three clinical variables, the two chemotherapy variables and one of the following groups of dosimetric features:

- Mean dose to the heart (1 variable if the whole-heart is considered; 5 variables if subparts are considered)
- Dose-volume indicators (24 or 24x5 variables)
- Dosiomics: first-order statistics (18 or 18x5 variables)
- Dosiomics: first-order statistics and texture features (93 or 93x5 variables).

We eliminate predictors that have the same values for every patient and those that are duplicates of another predictor in the sense that the correlation between them is 1 (which occurred, but rarely, for some of our train sets in the case where the heart subparts are considered).

Regarding the characterization of the first diagnosed cancer, we introduce 42 indicator variables, based on the International Classification of Childhood Cancer (33). An indicator variable is then kept if the association with CD occurrence is statistically significant (p-value*<* 0.01): the Chi-2 test is performed unless there are fewer than ten cases, in which case the Fisher test is preferred.

Note that the final number of included predictors in each model may vary due to the cross-validation: this pre-filtering step is performed independently on each train set.

##### 2.3.1.2 Clustering-based redundancy elimination

Due to the large number of features, we set a procedure to eliminate highly correlated features from dosiomics. Even if the machine learning algorithms might deal with correlated features, this helps the convergence of learning procedures. We perform hierarchical (agglomerative) clustering over the features with the complete-linkage function, which means that the distance between two clusters is the maximum distance between the points of the two clusters. The distance is 1 - Kendall’s tau, a rank correlation statistic. We keep clusters with a distance threshold of 0.2. This ensures that every pair of features that belongs to the same cluster has Kendall’s tau above 1 − 0.2 = 0.8. Then, for each cluster, the representative feature is selected by the highest hazard ratio from a multivariate Cox model adjusted on all the features’ cluster. If the features are extracted over the heart’s subparts, this hierarchical clustering step is performed over each subpart. See the **Supplementary Material** for an illustration of the procedure.

#### 2.3.2 Statistical models

In this work, survival analysis is performed: we estimate the survival function of patients for high-grade CD events adjusted on the dosimetric, chemotherapy, and clinical features. Two classes of models are considered, which results in four statistical models.

First, we consider the semi-parametric Cox Proportional Hazard (Cox PH) regression model (27), which is the standard model used in survival analysis. Given the predictors of a patient *i*, $X_{i}=\left(X_{i}^{1},\ldots ,X_{i}^{p}\right)$ , the hazard function has the form:


$$\text{λ}(t|X_{i})=\lambda_{0}\left(t\right)\exp\;(\beta^{⊤}X_{i}),\;\beta \in {\mathbb{R}}^{p}$$

The large number of predictors leads us to consider the Lasso penalty (Cox Lasso) (24) when maximizing the Cox’s partial likelihood for feature selection. The model is then re-adjusted, without the penalty term, using only the features with non-zero coefficients. The penalty is selected via a 5-fold cross-validation and is the largest penalty such that the corresponding error is within one standard deviation error of the minimum error (lambda.1se in the glmnetR package).

Another way of estimating a sparse number of coefficients with the Cox PH model is feature selection based on bootstrap sampling (Cox Bootstrap Lasso) (28). One hundred bootstrap samples are drawn from the train set. For each bootstrap sample, we fit a Cox Lasso model. We select the penalty by taking the largest one not rejected by a likelihood ratio test compared to the penalty that minimizes the error (the models are nested because a larger penalty implies a sparser model). Then, the selected features are stored. When the 100 bootstrap samples are fitted, a Cox model adjusted on the subset of features selected in above 90% of the bootstraps is fitted on the whole train set.

The second class of models is the Random Survival Forest (RSF), a non-parametric ensemble method based on survival trees. A Random Survival Forest contains *B* survival trees. Each survival tree learns from a bootstrap of the entire training data set and a subset of the predictors. Each survival tree separates the bootstrap into smaller groups of patients while maximizing the difference in survival curves between the groups. The risk prediction is then based on the survival trees’ predictions.

The four models’ hyper-parameters (Cox PH, Cox Lasso, Cox Bootstrap Lasso, RSF) are tuned with 5-fold cross-validation by maximizing the C-index. Once the hyper-parameters are tuned, we estimate the prediction errors.

#### 2.3.3 Prediction error estimation

The chosen prediction metrics are Harrell’s C-index, C-index corrected with inverse-probability-of-censoring weights (IPCW C-index), and the integrated Brier score over times from 1 to 60 years with a step of 1 year. As the models may have different predictors, we ensure that the IPCWs are estimated with the same subset of predictors based on clinical variables, except for the first diagnosed cancer. As Harrell’s C-index depends on the distribution of censoring times, we chose to estimate both C-index to show how the censor may influence the performance estimation.

These three metrics are estimated in a stratified 5-fold cross-validation procedure: the proportion of CD events is almost the same among the folds (about 5%). For each fold, Section 2.3.1 and Section 2.3.2 are run on the related train set, and the metrics are computed on the related test set.

After the 5-fold cross-validation, we estimate more precisely the models’ error via time-dependent error curves. We draw 100 bootstraps. For each time τ∈{1,2,…,60} years, we fit the models on the bootstrap, and we compute over the out-of-bag samples the Brier score *BS* (*τ*), and the bounded IPCW C-index *C_τ_* (34), which correspond to the IPCW C-index whose events that occurred above *τ* are discarded. Due to the large number of models, we select one representative model among Cox Lasso, Cox Bootstrap Lasso, and RSF based on their performance on the 5-fold cross-validation. We also run this procedure for the standard models (Cox with mean heart dose, Cox doses-volumes, Cox Lasso doses-volumes). The hyper-parameters are those which performed the best in the 5-fold cross-validation.

### 2.4 Tools

The study being computationally intensive, we used the HPC resources from the “Mésocentre” computing center of CentraleSupélec and École Normale Supérieure Paris-Saclay supported by CNRS and Région Île-de-France. Snakemake (35) was used to make the analyses consistent and reproducible. Dosiomics were extracted using pyradiomics (36). Machine learning models were performed in R with survival, glmnet and randomForestSRC. Results metrics were computed using the same calls of the pec package (37), but we developed our own implementation of error curves estimation with bootstrap, in order to better distribute the computations on the HPC.

## 3 Results

### 3.1 Summary statistics

**Table 1** shows the descriptive statistics of the cohort. The median survival time of the patients’ study is 30.2 years. Among them, 374 patients have experienced a CD with a grade above 3 (5%, which implies a very imbalanced data set), whose median survival time is lower (23.6 years). These patients have been significantly more treated by RT (75.7% vs 53.8%) and chemotherapy (89% vs 76.2%). Their hearts have been more irradiated than the entire cohort (median is 2.07 Gy vs 0.01 Gy; 75th percentile is 17.1 Gy vs 1.31 Gy). It suggests that the dose received by the heart has discriminative power for the prognosis of high-grade CDs, which is an expected result (3, 38).

**Table 1:** Characteristics of the selected patients from the entire FCCSS cohort and the patients diagnosed with CD of grade ≥ 3.

Factors	7368 FCCSS patients	374 patients diagnosed with a cardiac disease of grade ≥ 3
Sex		
Male	4059 (55.1%)	202 (54.0%)
Female	3309 (44.9%)	172 (46.0%)
Age at diagnosis of the first cancer		
Median	5	6
0-5 years	3969 (53.9%)	182 (48.7%)
6-10 years	1490 (20.2%)	80 (21.4%)
11-15 years	1572 (21.3%)	96 (25.7%)
*>* 15 years	337 (4.6%)	16 (4.3%)
Treatment for the first cancer		
Radiotherapy	4258 (53.8%)	266 (75.7%)
Chemotherapy	5759 (76.2%)	308 (89.0%)
Both	3236 (41.9%)	231 (66.3%)
Age at event/censorship occurrence		
Median	37	32
0-20 years	608 (8.3%)	95 (25.4%)
21-30 years	1628 (22.1%)	79 (21.1%)
31-40 years	2307 (31.3%)	111 (29.7%)
41-50 years	1926 (26.1%)	66 (17.6%)
*>* 50 years	899 (12.2%)	23 (6.1%)
Survival time		
Median	30.2	23.6
0-5 years	34 (0.5%)	32 (8.6%)
5-10 years	372 (5.0%)	29 (7.8%)
10-20 years	611 (8.3%)	85 (22.7%)
20-30 years	2615 (35.5%)	11 (29.7%)
30-40 years	2197 (29.8%)	80 (21.4%)
40-60 years	1478 (20.1%)	36 (9.6%)
*>* 60 years	61 (0.8%)	1 (0.3%)
Received dose to the heart (Gy)		
Min	0	0
25th percentile	0	0
Median	0.01	2.07
75th percentile	1.31	17.1
Max	47.8	47.8

### 3.2 Comparison of machine learning methods and groups of features

**Figure 2:**
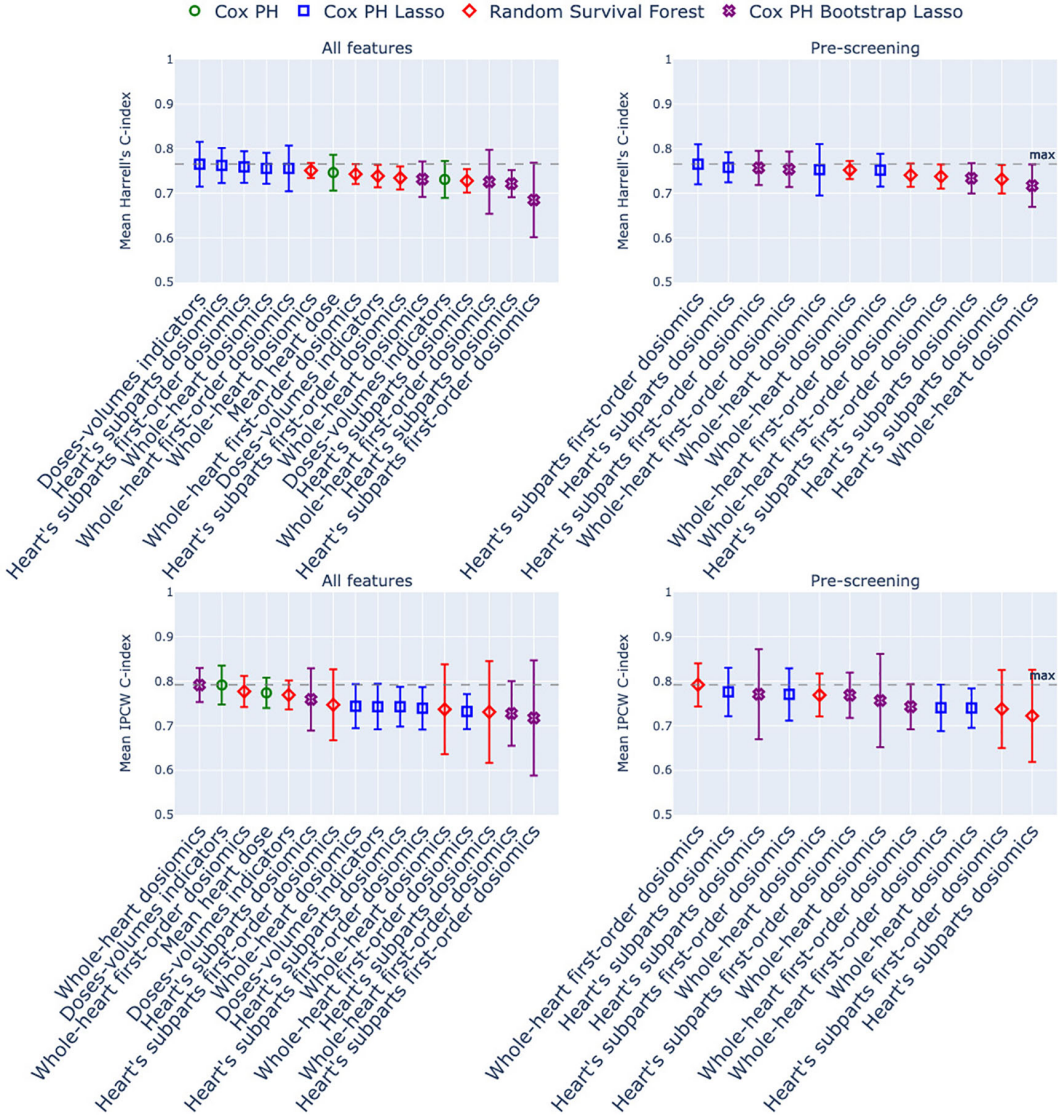
Harrell’s C-index and IPCW C-index of the models estimated with 5-fold stratified cross-validation. The x-axis corresponds to the group of dosimetric features used as predictors, and the marker/color corresponds to the statistical model. In green: Cox Proportional Hazard model; in blue: Cox with Lasso penalty; in purple: Cox with Bootstrap Lasso feature selection. Left column: no screening of correlated dosiomics; right column: screening of correlated dosiomics. The grey dotted line is the maximum C-index over the entire row.

**Figure 3:**
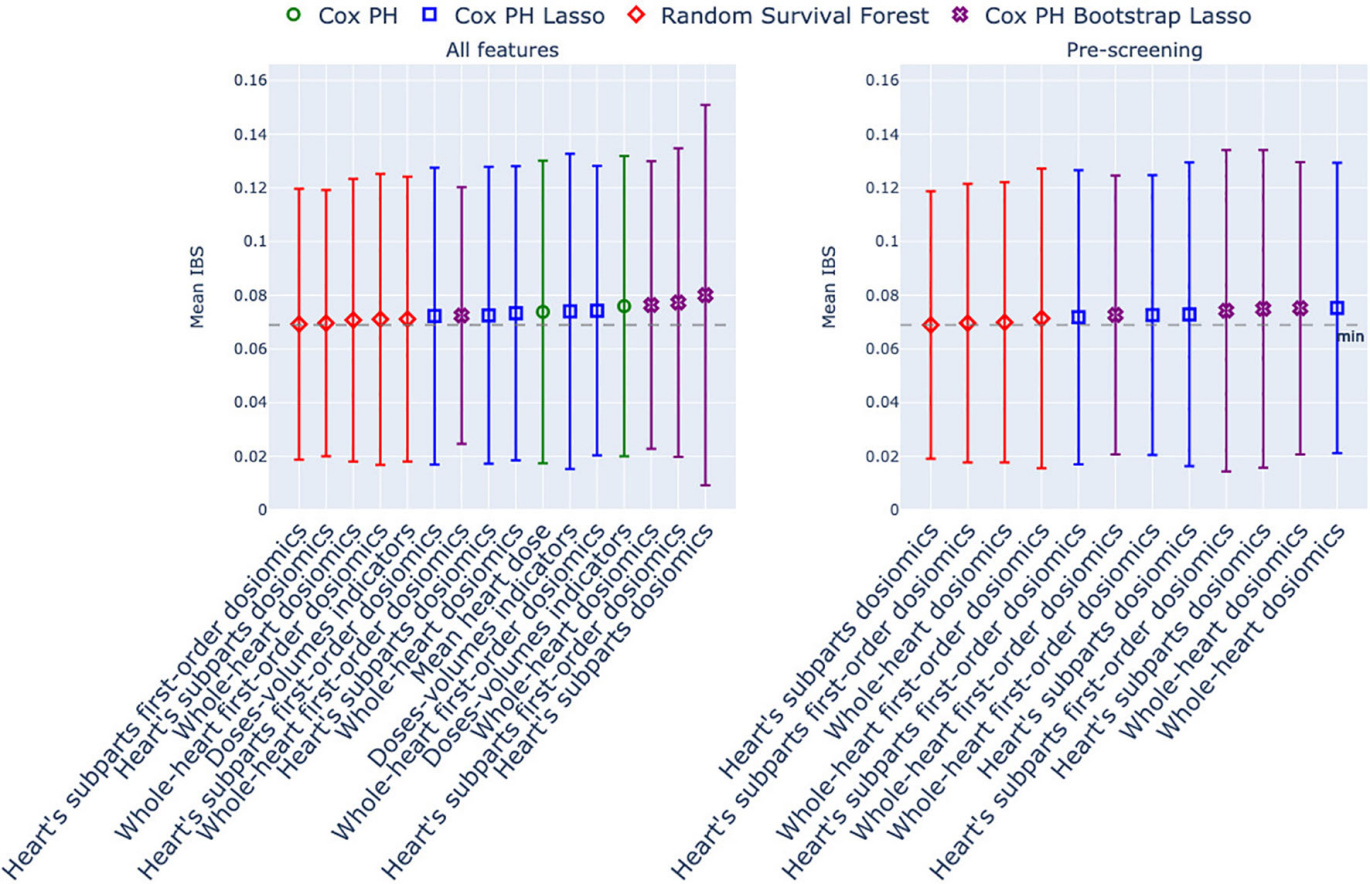
Integrated Brier Score of the models estimated with 5-fold stratified cross-validation. The x-axis corresponds to the group of dosimetric features used as predictors, and the marker/color corresponds to the statistical model. In green: Cox Proportional Hazard model; in blue: Cox with Lasso penalty; in purple: Cox with Bootstrap Lasso feature selection. Left column: no screening of correlated dosiomics; right column: screening of correlated dosiomics. The grey dotted line is the minimum IBS over the entire row.

This section presents the predictive performance estimation for the different models and groups of features mentioned in Section 2.3. **Figures 2** and **3** show the Harrell’s, IPCW C-index, and the Integrated Brier score distributions over the 5-fold cross-validation. The numerical results are reported in **Table 2**.

**Table 2:** Results of the stratified 5-fold cross-validation.

Model	All features			Pre-screening		
	Harrell’s C	IPCW C	IBS	Harrell’s C	IPCW C	IBS
Cox Mean heart dose	0.747 ± 0.040	0.774 ± 0.034	0.074 ± 0.056			
Cox Dose-volume indicators	0.731 ± 0.041	0.791 ± 0.044	0.076 ± 0.056			
Cox Lasso Dose-volume indicators	**0**.**765** ± **0**.**050**	0.743 ± 0.051	0.074 ± 0.059			
RSF Dose-volume indicators	0.739 ± 0.025	0.769 ± 0.033	0.071 ± 0.053			
Cox Lasso Whole heart first order dosiomics	0.756 ± 0.051	0.732 ± 0.039	0.074 ± 0.054	0.752 ± 0.037	0.740 ± 0.052	0.073 ± 0.052
Cox Lasso Heart’s subparts first order dosiomics	0.759 ± 0.035	0.739 ± 0.048	0.072 ± 0.055	**0**.**765** ± **0**.**045**	0.770 ± 0.059	0.072 ± 0.055
Cox Lasso Whole heart dosiomics	0.756 ± 0.035	0.744 ± 0.049	0.073 ± 0.055	0.753 ± 0.058	0.740 ± 0.044	0.075 ± 0.054
Cox Lasso Heart’s subparts dosiomics	0.762 ± 0.039	0.742 ± 0.045	0.073 ± 0.055	0.758 ± 0.034	0.776 ± 0.054	0.073 ± 0.057
Cox Bootstrap Lasso Whole heart first order dosiomics	0.726 ± 0.072	0.727 ± 0.072	0.072 ± 0.048	0.754 ± 0.040	0.743 ± 0.051	0.073 ± 0.052
Cox Bootstrap Lasso Heart’s subparts first order dosiomics	0.685 ± 0.084	0.717 ± 0.129	0.077 ± 0.057	0.757 ± 0.039	0.769 ± 0.051	0.074 ± 0.060
Cox Bootstrap Lasso Whole heart dosiomics	0.732 ± 0.040	**0**.**791** ± **0**.**038**	0.076 ± 0.054	0.717 ± 0.048	0.756 ± 0.105	0.075 ± 0.054
Cox Bootstrap Lasso Heart’s subparts dosiomics	0.721 ± 0.030	0.759 ± 0.070	0.080 ± 0.071	0.734 ± 0.034	0.771 ± 0.101	0.075 ± 0.059
RSF Whole heart first order dosiomics	0.743 ± 0.022	0.777 ± 0.035	0.071 ± 0.054	0.741 ± 0.026	**0**.**792** ± **0**.**049**	0.071 ± 0.056
RSF Heart’s subparts first order dosiomics	0.734 ± 0.026	0.747 ± 0.080	**0**.**069** ± **0**.**050**	0.738 ± 0.027	0.738 ± 0.088	0.070 ± 0.052
RSF Whole heart dosiomics	0.751 ± 0.017	0.737 ± 0.101	0.071 ± 0.053	0.752 ± 0.020	0.769 ± 0.048	0.070 ± 0.052
RSF Heart’s subparts dosiomics	0.728 ± 0.026	0.731 ± 0.114	0.070 ± 0.050	0.732 ± 0.032	0.722 ± 0.103	**0**.**069** ± **0**.**050**

The format is: mean ± standard deviation. In bold: the best score of the corresponding column (maximum for C-index, minimum for IBS).

The three indices generate different model rankings. The IBS is the most stable index in average, and all the models display a large and constant inter-fold variability (**Figure 3**). For the Harell’s C-index (**Figure 2**, above row), the top-ranked models are all Cox Lasso models, whether or not a screening stage is included. In contrast, for the IPCW C-index, three other models stand out: Cox Bootstrap Lasso with dosiomics extracted from the whole heart, Cox with dose-volume indicators, and Random Survival Forest with screened first-order dosiomics extracted on the whole heart. Overall, we can observe that, whatever the indices used for the comparison, no model outperforms the others: the mean error differences are not outstanding; **Figures 2** and **3** show that most mean errors are above the mean minus the standard error of the first-ranked model.

We now select the standard models (Cox with mean heart dose, Cox with dose-volume indicators), plus three models of different types (Cox Lasso, Cox Bootstrap Lasso, RSF) that performed best regarding the IPCW C-index within their own model group for deeper performance estimation. First, we confirmed that none of these three models were statistically different, in terms of their C-index mean estimations, from the Cox model adjusted on the mean dose to the heart: **Table 3** reports the p-values of Wilcoxon’s tests (U-test) conducted on the IPCW C-indexes from the stratified cross-validation over these three models against the Cox PH mean dose to the heart. None is below the significant threshold of 0.05. Therefore, we cannot assess the statistical difference between the C-index mean estimations and the statistical significance between one of the three best models and the Cox model adjusted on the mean dose to the heart.

**Table 3:** P-values of the Wilcoxon’s test run over the IPCW C-indexes of the best Random Survival Forest, Cox Lasso, and Cox Bootstrap Lasso models against the Cox mean heart dose model.

Model	Random Survival Forest first-order whole-heart dosiomics with pre-screening	Cox Lasso heart’s subparts dosiomics with pre-screening	Cox Bootstrap Lasso whole heart dosiomics
Cox mean heart dose	0.69	1.0	0.79

Second, in order to better understand the reasons of these similar performances, we investigated on the variables selected in the Cox Lasso model adjusted on the first-order dosiomics and dose-volume indicators (see the **Supplementary Material** for the Cox’s coefficients estimated on each fold for both models). The main selected dosimetric features for the model adjusted on the first-order dosiomics are the mean, the median of the 10%-quantile of the dose distribution, whereas *D*_70_ (30%-quantile of the dose distribution) and *V*_2_ (volume percentage irradiated above 2 Gy) are the most significant ones for the dose-volume indicators’ model.

**Figure 4:**
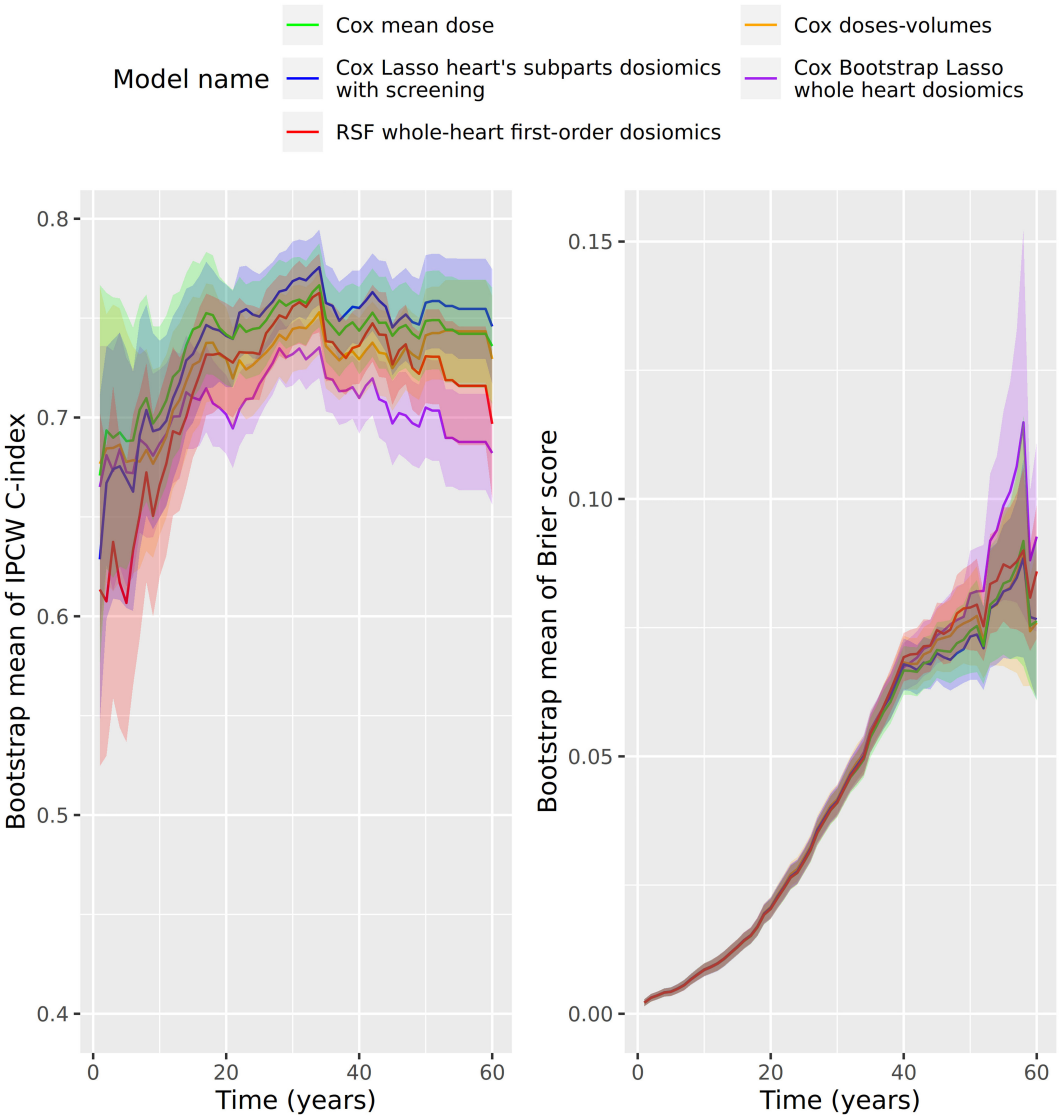
Error prediction in function of time (years) of the best model of each statistical model type (Cox Lasso, Cox Bootstrap Lasso, RSF), plus the Cox models adjusted on the mean dose to the heart and dose-volume indicators. On the left: IPCW-C-index; On the right: Brier score.

Third, we estimate the time-dependent error curves as described in Section 2.3.3. **Figure 4** shows the time-dependent C-index and Brier score over 60 years. First, the Brier score is very stable until 40 years. Most of the variation comes from Brier scores between 40 and 60 years. Patients’ survival times included in this range represents less than 10% of the cohort (**Table 1**). However, there are some differences in the predictive performance regarding the IPCW C-index. The Cox PH with mean heart dose model outperforms the models from 0 to 20 years, but the Cox Lasso with screened heart’s subparts dosiomics performs better between 20 and 60 years. Also, there is variability in the C-index estimation over the whole time scale (**Figure 4**). The Cox Bootstrap Lasso was the first ranked model in the 5-fold stratified cross-validation, but the model is the lowest ranked with the 100 bootstrap samples error estimation.

## 4 Discussion

In this study, we explored the benefits, in terms of predictive performance, of dosiomics compared to standard dosimetric features, with the help of machine learning methods, for the prognosis of high-grade CD occurrence in childhood cancer survivors. We performed survival analysis, adapted to censored data, which avoids the bias of discarding patients on a large multi-centric cohort with a very long follow-up period. Efforts were made to estimate the statistical uncertainty of our models. First, we used resampling methods (cross-validation, bootstrap sampling) to assess how well the models are generalizable. Second, we used global and time-dependent metrics in our study; since the distribution of survival times is large (see **Table 1**), high-grade CD may occur late.

Several difficulties have been addressed. First, the large number of patients, models, and resampling methods have made the study computationally intensive. The preprocessing of 3D dose matrices and statistical learning computations have been well organized and distributed over an HPC cluster. The high censorship of the dataset (5% of high-grade CD) might also harm the statistical learning. Combining this with the many statistical model fits may imply convergence issues, and routines have been designed robustly to ensure the convergence of each model’s fit.

To our knowledge, this is the first application of dosiomics for risk estimation of high-grade CD in childhood cancer survivors. Dosiomics have been mainly used to predict radiation pneumotisis (13, 15, 16, 20, 21, 39, 40), but also other pathologies such as head and neck cancers (17) (see (14) for other examples). Our study confirms the RT-induced late effect of high-grade CD in childhood cancer survivors (41).

Since there is no comparable case study of dosiomics for high-grade CD prognosis, it is difficult to quantitatively compare our results with other studies. Indeed, studies either consider another clinical outcome or much smaller cohorts, perform classification instead of survival analysis, have a different strategy for estimating the statistical generalization or integrate radiomics of CT-scans (17, 20, 21, 39, 40, 42, 43). We found that dosiomics were not statistically significant in terms of global metrics (see Section 3.2) compared to standard models based on dosimetric features. Indeed, p-values (**Table 3**) are not below the threshold 0.05, which imply we cannot reject the hypotheses that mean estimations of C-indexes are the same. In terms of the Brier score, the models have similar performance. There are slightly more variations of the C-indexes, both with or without censoring weight correction, but no dosiomics model has a statistically better performance than the standard ones. An interesting result is that the variables selected by the dosiomics models are the mean, the median of the 10%-quantile of the dose distribution, i.e. variables that contain globally the same information as the dose-volume histograms. It would tend to indicate that, for risk prediction purpose, a description of the dose distribution by the dose-volume histograms could be sufficient. Note that the variables selected by the dose-volume models are *D*_70_ and *V*_2_.

In the literature, dosiomics are often combined with radiomics for better performance (18). In specific cases, dosiomics-based models do not perform better than standard methods alone, but the combination of radiomics and dosiomics does (21, 39, 40). These unavailable CT-scans in our study may explain a lack of additional predictive performance compared to dose-volume indicators. Note that genetic interactions with dosiomics have also been explored for example for lung cancer (44).

However, some dosiomics models might have better predictive performance in specific time ranges, as shown by the time-dependent error curves (**Figure 4**). In terms of medical monitoring, it is essential to assess the models performances at different time scales for patient care improvement, since this cohort study spreads over time. To our knowledge, this has not been much discussed in dosiomics-based survival analysis studies.

We focused on prognosis performance in this study, mainly having the medical monitoring context in mind. However, dosiomics may be helpful in another context, such as the stability of feature extraction under dose distribution reconstruction error (45). Also, note that accessing the voxelized dose leads to an improved mean estimation of the received dose to the heart (30), which is stable across various dose distributions, techniques and centers (46), supporting the assertion that obtaining voxelized data is meaningful.

## 5 Conclusion

Regarding global metrics, dosiomics-based models do not significantly outperform the prognosis performance of standard models in the case of the late risk estimation of high-grade CDs in childhood cancer survivors. Quantiles of the dose distribution, given by dose-volume indicators or first-order dosiomics, summarize the information contained in the dose distribution for the prognosis of RT-induced severe CDs. The numerous models considered in this study may have performance differences for specific periods, which is attractive regarding the medical monitoring of late effects. As the exploration of dosiomics emerges in oncology, assessing the robustness and generalization of such methods with various use cases is crucial.

## Data Availability

The data analyzed in this study is subject to the following licenses/restrictions: The dataset is not publicly available. Requests to access these datasets should be directed to rodrigue.allodji@gustaveroussy.fr.
