# Supplementary material for: Combining dosiomics and machine learning methods for predicting severe cardiac diseases in childhood cancer survivors: the French Childhood Cancer Survivor Study
Source: Front Oncol. 2024 Dec 2;14:1241221. doi: 10.3389/fonc.2024.1241221 (PMC11647004; doi:10.3389/fonc.2024.1241221)
Supplement: Supplementary file 1 [file DataSheet1.pdf]

Supplementary Material

1 SUPPLEMENTARY TABLES AND FIGURES

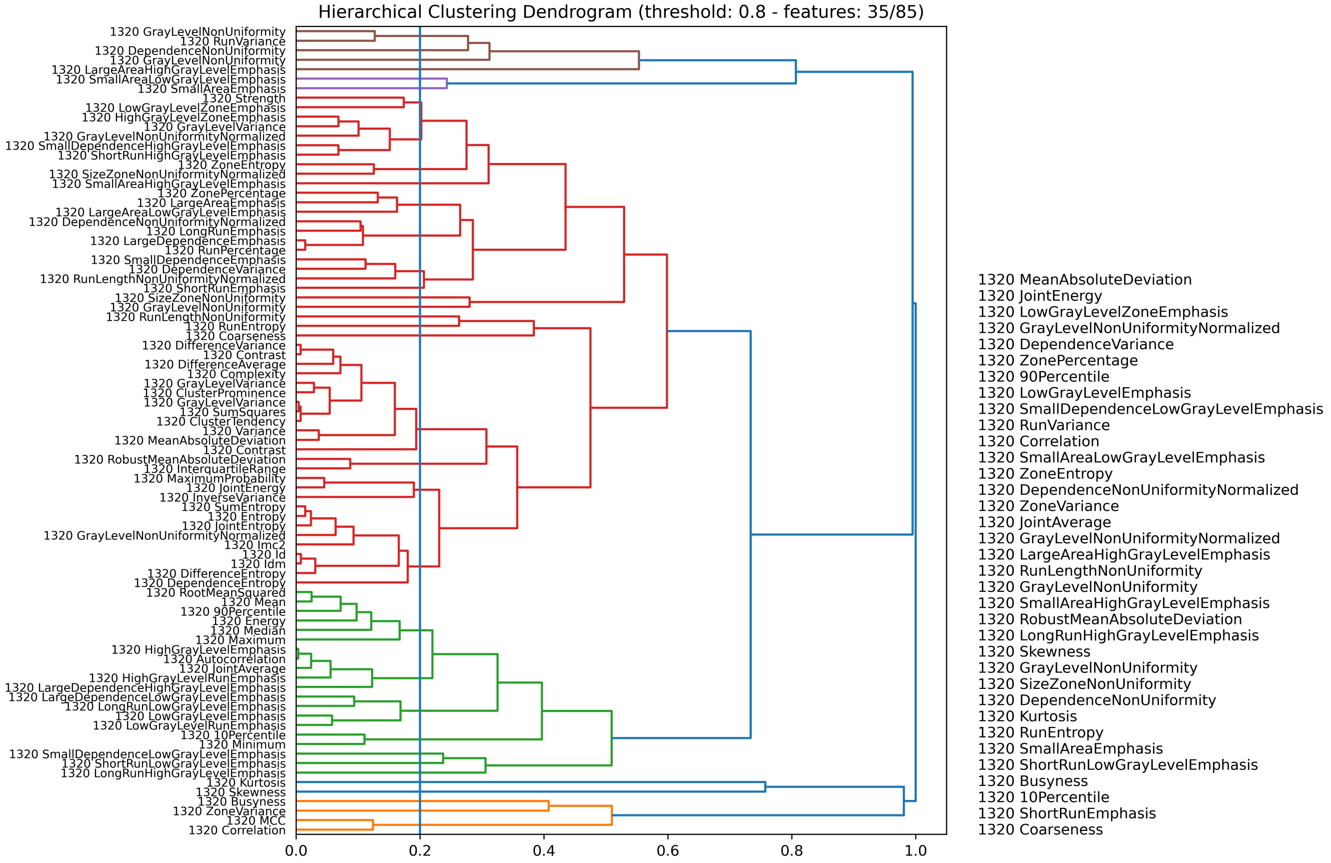

Figure S1. Illustration of the clustering-based redundancy elimination. Hierarchical clustering is performed with the distance 1 - Kendall's tau over the dosiomics extracted from the whole heart, with a threshold of 0.8. We select the best feature among each cluster by performing a multivariate Cox adjusted on the cluster's features. The selected features are shown on the right side. In this procedure, 35 features were kept over 85.

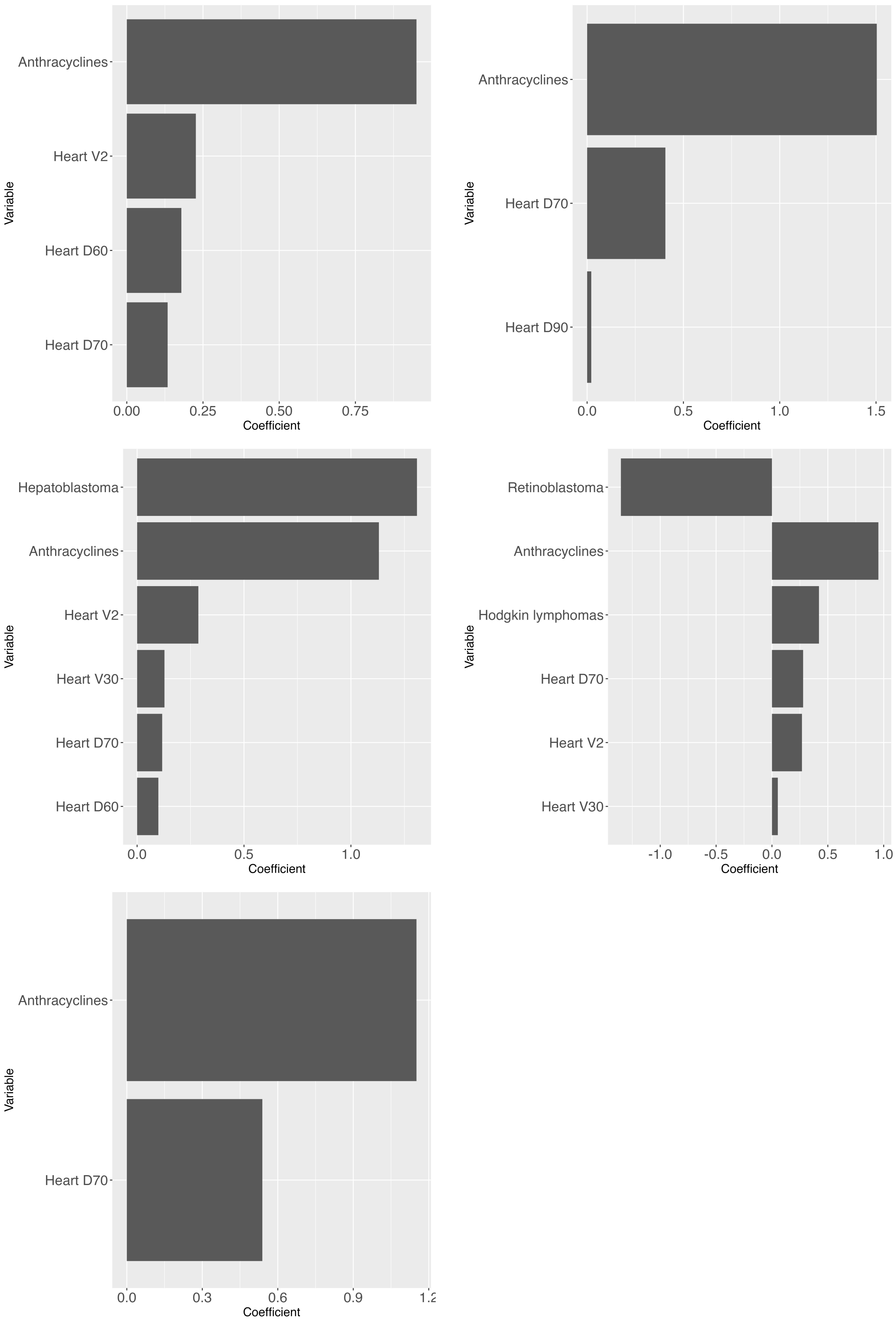

Figure S2. Model: Cox Lasso adjusted on the dose-volume indicators. Coefficients of the variables selected by the Lasso for each fold of the 5-fold cross-validation.

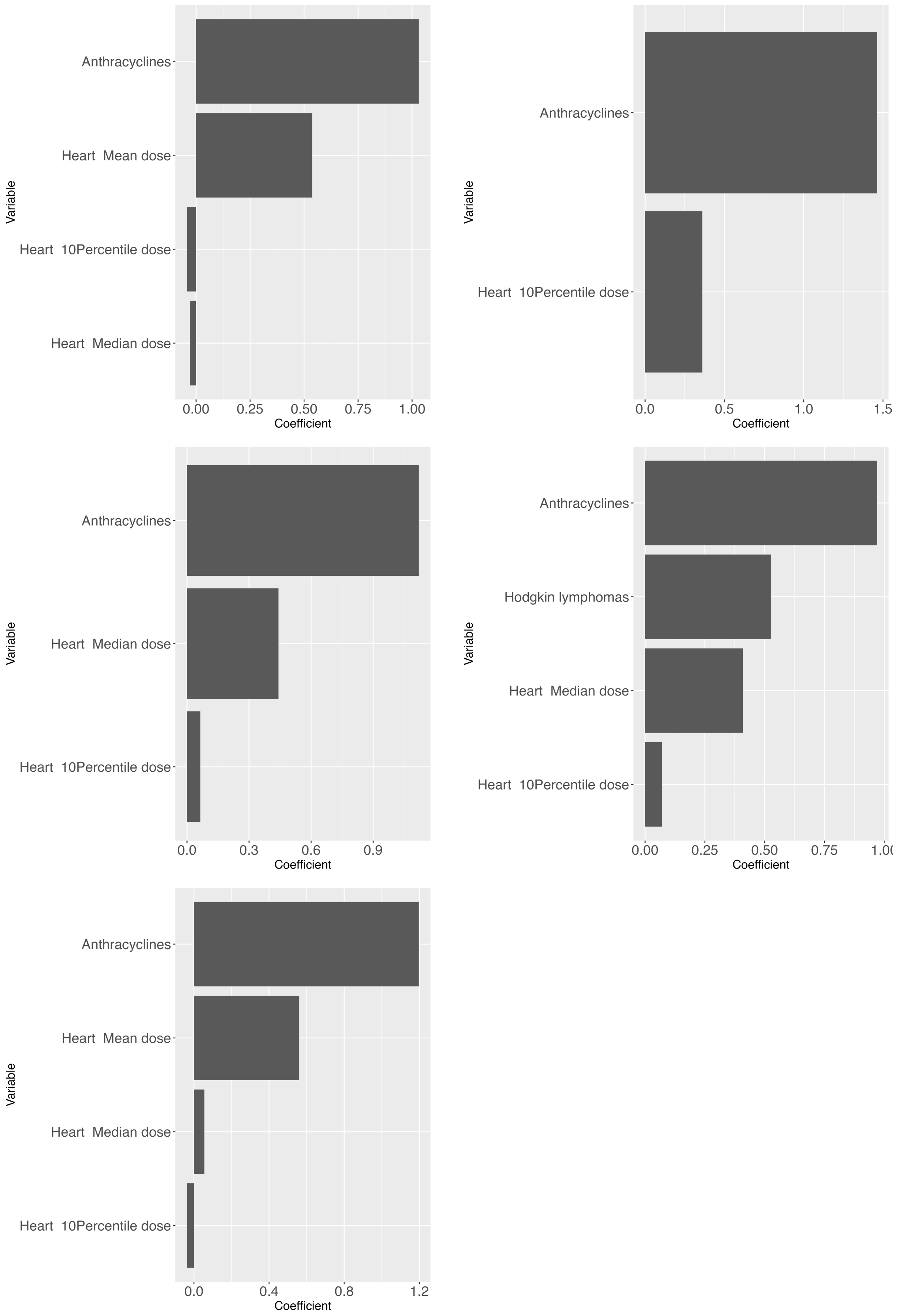

Figure S3. Model: Cox Lasso adjusted on the first-order dosiomics. Coefficients of the variables selected by the Lasso for each fold of the 5-fold cross-validation.
